# Tetra­aqua­bis(3,5-di-4-pyridyl-1,2,4-triazolato-κ*N*)nickel(II) dihydrate

**DOI:** 10.1107/S1600536809027688

**Published:** 2009-07-22

**Authors:** Lin Yi Dong

**Affiliations:** a School of Pharmacy, Tianjin Medical University, Tianjin 300070, People’s Republic of China

## Abstract

The Ni^II^ atom in the title compound, [Ni(C_12_H_8_N_5_)_2_(H_2_O)_4_]·2H_2_O, lies on a center of inversion and is coordinated by the N atoms of two 3,5-di-4-pyridine-1,2,4-triazolate ligands and by four water O atoms in a slightly distorted octa­hedral geometry. The coordinated and uncoordinated water mol­ecules inter­act with the *N*-heterocycles through O—H⋯N and O—H⋯O hydrogen bonds, generating a three-dimensional supra­molecular architecture.

## Related literature

For magnetic studies of transition metal complexes with 1,2,4- triazole derivatives, see: Haasnoot (2000 ▶). For 3,5-di-4-pyridine-1,2,4-triazole, see: Zhang *et al.* (2005 ▶, 2006 ▶). For the synthesis, see: Basu & Dutta (1964 ▶). 
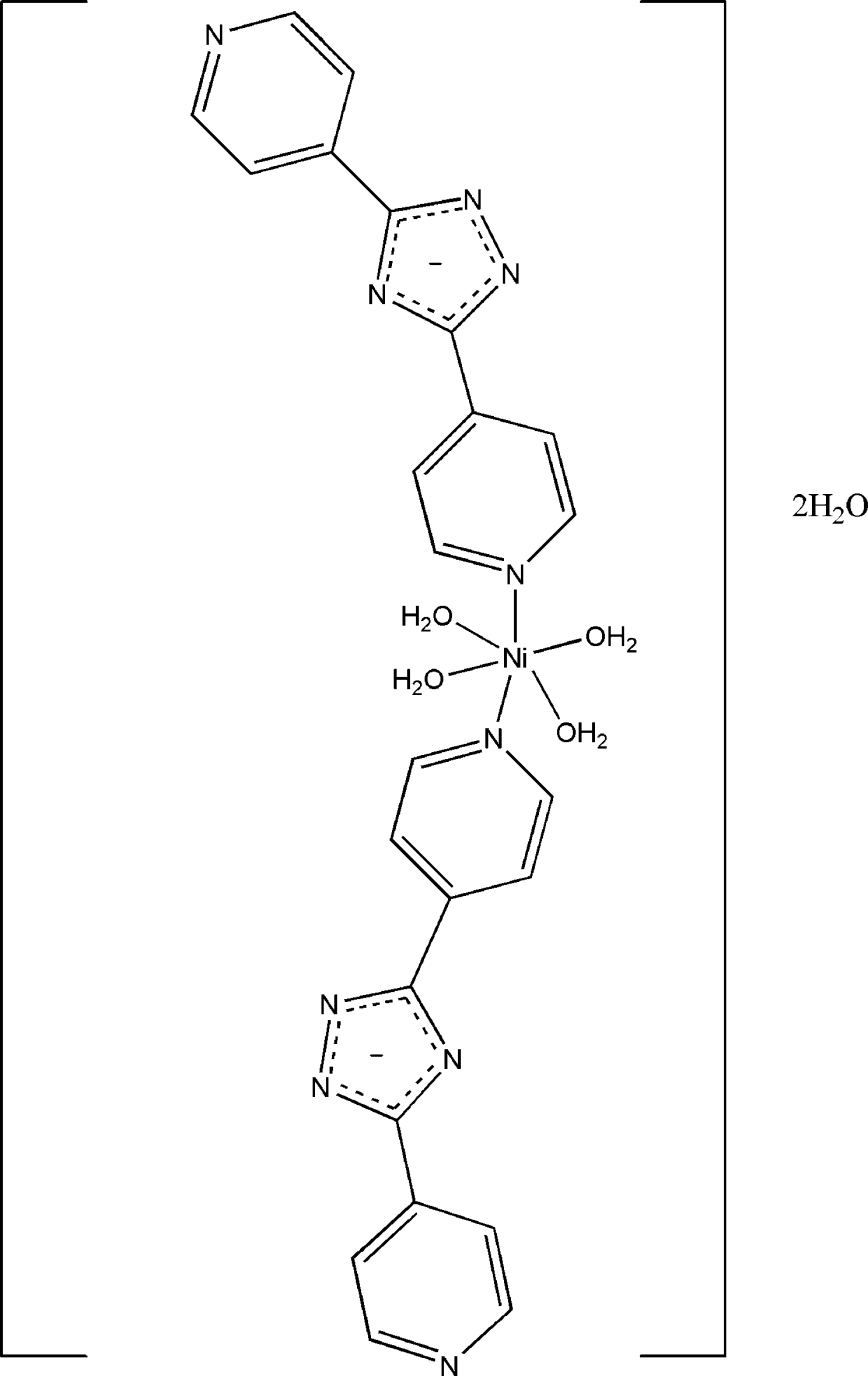


## Experimental

### 

#### Crystal data

[Ni(C_12_H_8_N_5_)_2_(H_2_O)_4_]·2H_2_O
*M* *_r_* = 611.27
Monoclinic, 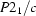

*a* = 7.3390 (15) Å
*b* = 15.653 (3) Å
*c* = 11.829 (2) Å
β = 107.20 (3)°
*V* = 1298.1 (5) Å^3^
*Z* = 2
Mo *K*α radiation
μ = 0.81 mm^−1^
*T* = 293 K
0.43 × 0.27 × 0.21 mm

#### Data collection

Bruker SMART CCD area-detector diffractometer
Absorption correction: multi-scan (*SADABS*; Sheldrick, 1996 ▶) *T*_min_ = 0.722, *T*_max_ = 0.848
10883 measured reflections
2344 independent reflections
2131 reflections with *I* > 2σ(*I*)
*R*_int_ = 0.034

#### Refinement

*R*[*F* ^2^ > 2σ(*F* ^2^)] = 0.036
*wR*(*F* ^2^) = 0.078
*S* = 1.14
2344 reflections
211 parameters
H atoms treated by a mixture of independent and constrained refinement
Δρ_max_ = 0.26 e Å^−3^
Δρ_min_ = −0.34 e Å^−3^

### 

Data collection: *SMART* (Bruker, 1998 ▶); cell refinement: *SAINT* (Bruker, 1999 ▶); data reduction: *SAINT*; program(s) used to solve structure: *SHELXTL* (Sheldrick, 2008 ▶); program(s) used to refine structure: *SHELXTL*; molecular graphics: *SHELXTL*; software used to prepare material for publication: *SHELXTL*.

## Supplementary Material

Crystal structure: contains datablocks I, global. DOI: 10.1107/S1600536809027688/hg2531sup1.cif

Structure factors: contains datablocks I. DOI: 10.1107/S1600536809027688/hg2531Isup2.hkl

Additional supplementary materials:  crystallographic information; 3D view; checkCIF report

## Figures and Tables

**Table 1:** Hydrogen-bond geometry (Å, °)

*D*—H⋯*A*	*D*—H	H⋯*A*	*D*⋯*A*	*D*—H⋯*A*
O2—H2*A*⋯O3^i^	0.82 (3)	2.03 (3)	2.818 (3)	160 (3)
O3—H3*A*⋯N4^ii^	0.85 (3)	2.09 (3)	2.939 (3)	172 (3)
O3—H3*B*⋯N5^iii^	0.86 (4)	1.94 (4)	2.789 (3)	173 (3)

Symmetry codes: (i) 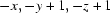; (ii) 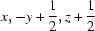; (iii) 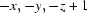.

**Fig. 1.**
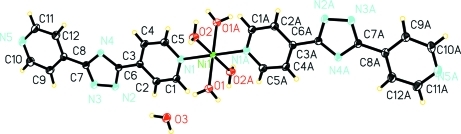
View of the title compound, with displacement ellipsoids drawn at the 40% probability level.

## supplementary crystallographic information

### Comment

Transition metal complexes with 1,2,4-triazole derivatives as ligands are of great interest as they are the subject of magnetic studies (Haasnoot, 2000). The ligand 3,5-di(4-pyridine)-1,2,4-triazole is of special interest as it contains multi-dentate donor atoms and shows diverse coordination modes. Especially only a few examples about the coordinaiton chemistry of *L* are reported. Some unusual coordination modes of *L* also have been reported forming interesting supramolecular isomerism systems (Zhang *et al.*, 2006).

In this work, we synthesized a new compound [Ni(*L*)_2_(H_2_O)_4_](H_2_O)_2_ (*L* = 3,5-di(4-pyridine)-1,2,4-triazolate anions), which is composed of one nickel(II) cation, two *L* ligand, four coordinated and two lattice water molecules. The nickel(II) cation is six-coordinated in the octahedral geometry. The equatorial site of nickel cation is occupied by four aqua molecules while the axial site is occupied by two nitrogen atoms of two mono-dentate *L* ligands. The mono-dentate coordination mode of *L* is different from previously reported di-, tri- or tetra-dentate coordination modes of *L* (Zhang *et al.*, 2005; Zhang *et al.*, 2006).

O3 acts as both a hydrogen bond donor and a hydrogen bond acceptor. Strong O—H···N and O—H···O hydrogen bonds generated from water molecules and nitrogen atoms of pyridine or triazole groups are also observed resulting in the three-dimensional supramolecular network.

### Experimental

The ligand was prepared according to the previous literature (Basu & Dutta, (1964)). [Ni(*L*)_2_(H_2_O)_4_](H_2_O) (I) (*L* = 3,5- di(4-pyridine)-1,2,4-triazole) was prepared under the hydrothermal conditions. [Ni(ClO_4_)_2_].6H_2_O (0.2 mmol), *L* (0.2 mmol) and 18 ml water was added to a 25 ml reaction vessel. The reaction vessel was then sealed and subsequently placed in an oven for 140 h at 160°C well shaped green block crystals were obtained and washed with ethanol.

### Refinement

All H atoms were found on difference maps. The water H atoms were refined freely, giving an O–H = 0.82–0.86 Å. The remaining H atoms were placed in calculated positions, with C–H = 0.93 Å, and included in the final cycles of refinement using a riding model, with *U*_iso_(H) = 1.2*U*_eq_(C)

### Crystal data

**Table:**

[Ni(C_12_H_8_N_5_)_2_(H_2_O)_4_]·2H_2_O	*F*(000) = 636
*M**_r_* = 611.27	*D*_x_ = 1.564 Mg m^−^^3^
Monoclinic, *P*2_1_/*c*	Mo *K*α radiation, λ = 0.71073 Å
Hall symbol: -P 2ybc	Cell parameters from 467 reflections
*a* = 7.3390 (15) Å	θ = 1.5–25.3°
*b* = 15.653 (3) Å	µ = 0.81 mm^−^^1^
*c* = 11.829 (2) Å	*T* = 293 K
β = 107.20 (3)°	Block, green
*V* = 1298.1 (5) Å^3^	0.43 × 0.27 × 0.21 mm
*Z* = 2	

### Data collection

**Table:**

Bruker SMART CCD area-detector diffractometer	2344 independent reflections
Radiation source: fine-focus sealed tube	2131 reflections with *I* > 2σ(*I*)
graphite	*R*_int_ = 0.034
φ and ω scans	θ_max_ = 25.2°, θ_min_ = 3.2°
Absorption correction: multi-scan (*SADABS*; Sheldrick, 1996)	*h* = −8→8
*T*_min_ = 0.722, *T*_max_ = 0.848	*k* = −18→18
10883 measured reflections	*l* = −14→14

### Refinement

**Table:**

Refinement on *F*^2^	Primary atom site location: structure-invariant direct methods
Least-squares matrix: full	Secondary atom site location: difference Fourier map
*R*[*F*^2^ > 2σ(*F*^2^)] = 0.036	Hydrogen site location: inferred from neighbouring sites
*wR*(*F*^2^) = 0.078	H atoms treated by a mixture of independent and constrained refinement
*S* = 1.14	*w* = 1/[σ^2^(*F*_o_^2^) + (0.0259*P*)^2^ + 1.0454*P*] where *P* = (*F*_o_^2^ + 2*F*_c_^2^)/3
2344 reflections	(Δ/σ)_max_ < 0.001
211 parameters	Δρ_max_ = 0.26 e Å^−^^3^
0 restraints	Δρ_min_ = −0.34 e Å^−^^3^

### Special details

**Table:**

Geometry. All e.s.d.'s (except the e.s.d. in the dihedral angle between two l.s. planes) are estimated using the full covariance matrix. The cell e.s.d.'s are taken into account individually in the estimation of e.s.d.'s in distances, angles and torsion angles; correlations between e.s.d.'s in cell parameters are only used when they are defined by crystal symmetry. An approximate (isotropic) treatment of cell e.s.d.'s is used for estimating e.s.d.'s involving l.s. planes.
Refinement. Refinement of *F*^2^ against ALL reflections. The weighted *R*-factor *wR* and goodness of fit *S* are based on *F*^2^, conventional *R*-factors *R* are based on *F*, with *F* set to zero for negative *F*^2^. The threshold expression of *F*^2^ > σ(*F*^2^) is used only for calculating *R*-factors(gt) *etc*. and is not relevant to the choice of reflections for refinement. *R*-factors based on *F*^2^ are statistically about twice as large as those based on *F*, and *R*- factors based on ALL data will be even larger.

### Fractional atomic coordinates and isotropic or equivalent isotropic displacement parameters (Å^2^)

**Table:**

	*x*	*y*	*z*	*U*_iso_*/*U*_eq_	
C1	0.4927 (3)	0.33129 (14)	0.6238 (2)	0.0265 (5)	
H1C	0.5402	0.3645	0.6914	0.032*	
C2	0.4672 (3)	0.24522 (14)	0.6376 (2)	0.0272 (5)	
H2C	0.4983	0.2216	0.7131	0.033*	
C3	0.3951 (3)	0.19348 (13)	0.53900 (19)	0.0218 (5)	
C4	0.3570 (4)	0.23293 (14)	0.4292 (2)	0.0287 (5)	
H4A	0.3117	0.2010	0.3603	0.034*	
C5	0.3864 (4)	0.31953 (14)	0.4228 (2)	0.0283 (5)	
H5A	0.3590	0.3446	0.3483	0.034*	
C6	0.3545 (3)	0.10302 (13)	0.55253 (19)	0.0217 (5)	
C7	0.2505 (3)	−0.02231 (13)	0.52360 (19)	0.0224 (5)	
C8	0.1688 (3)	−0.10334 (14)	0.46845 (19)	0.0232 (5)	
C9	0.1536 (4)	−0.17397 (16)	0.5368 (2)	0.0370 (6)	
H9A	0.1892	−0.1697	0.6189	0.044*	
C10	0.0858 (4)	−0.24977 (16)	0.4823 (2)	0.0411 (7)	
H10A	0.0794	−0.2962	0.5301	0.049*	
C11	0.0428 (4)	−0.19348 (16)	0.3005 (2)	0.0359 (6)	
H11A	0.0042	−0.1995	0.2186	0.043*	
C12	0.1111 (4)	−0.11487 (15)	0.3471 (2)	0.0312 (6)	
H12A	0.1184	−0.0699	0.2971	0.037*	
H1A	0.413 (5)	0.550 (2)	0.686 (3)	0.051 (9)*	
H2A	0.149 (5)	0.527 (2)	0.358 (3)	0.051 (10)*	
H3A	0.163 (5)	0.426 (2)	0.782 (3)	0.054 (10)*	
H1B	0.283 (5)	0.493 (2)	0.633 (3)	0.055 (10)*	
H2B	0.257 (4)	0.5122 (18)	0.292 (3)	0.048 (9)*	
H3B	0.077 (5)	0.372 (2)	0.691 (3)	0.062 (10)*	
N1	0.4525 (3)	0.36961 (11)	0.51811 (16)	0.0237 (4)	
N2	0.3943 (3)	0.06670 (12)	0.65942 (16)	0.0279 (5)	
N3	0.3259 (3)	−0.01470 (12)	0.64046 (17)	0.0290 (5)	
N4	0.2645 (3)	0.04985 (11)	0.46332 (16)	0.0226 (4)	
N5	0.0286 (3)	−0.26126 (13)	0.3656 (2)	0.0360 (5)	
Ni1	0.5000	0.5000	0.5000	0.01983 (13)	
O1	0.3476 (3)	0.53202 (12)	0.61834 (15)	0.0275 (4)	
O2	0.2507 (3)	0.50476 (11)	0.35989 (15)	0.0287 (4)	
O3	0.1179 (3)	0.42339 (11)	0.70636 (17)	0.0314 (4)	

### Atomic displacement parameters (Å^2^)

**Table:**

	*U*^11^	*U*^22^	*U*^33^	*U*^12^	*U*^13^	*U*^23^
C1	0.0368 (14)	0.0208 (12)	0.0204 (12)	−0.0048 (10)	0.0062 (10)	−0.0025 (9)
C2	0.0390 (14)	0.0215 (12)	0.0197 (12)	−0.0036 (10)	0.0066 (10)	0.0023 (9)
C3	0.0249 (12)	0.0178 (11)	0.0234 (12)	−0.0009 (9)	0.0083 (9)	−0.0001 (9)
C4	0.0434 (15)	0.0199 (12)	0.0204 (12)	−0.0063 (11)	0.0057 (10)	−0.0029 (9)
C5	0.0416 (15)	0.0216 (12)	0.0199 (12)	−0.0021 (10)	0.0062 (10)	0.0026 (9)
C6	0.0269 (12)	0.0174 (11)	0.0209 (11)	−0.0006 (9)	0.0073 (9)	0.0001 (9)
C7	0.0281 (12)	0.0162 (11)	0.0224 (11)	−0.0009 (9)	0.0068 (9)	−0.0005 (8)
C8	0.0239 (12)	0.0188 (11)	0.0254 (12)	−0.0003 (9)	0.0051 (9)	−0.0010 (9)
C9	0.0588 (18)	0.0260 (13)	0.0251 (13)	−0.0116 (12)	0.0108 (12)	0.0001 (10)
C10	0.0624 (19)	0.0225 (13)	0.0392 (16)	−0.0129 (13)	0.0161 (13)	0.0020 (11)
C11	0.0424 (15)	0.0314 (14)	0.0280 (13)	−0.0042 (11)	0.0014 (11)	−0.0060 (11)
C12	0.0400 (15)	0.0221 (12)	0.0269 (13)	−0.0029 (10)	0.0031 (11)	0.0039 (10)
N1	0.0310 (11)	0.0168 (9)	0.0223 (10)	−0.0024 (8)	0.0067 (8)	−0.0006 (7)
N2	0.0413 (12)	0.0175 (10)	0.0232 (10)	−0.0064 (9)	0.0070 (9)	0.0002 (8)
N3	0.0429 (12)	0.0189 (10)	0.0232 (10)	−0.0071 (9)	0.0067 (9)	0.0009 (8)
N4	0.0281 (10)	0.0161 (9)	0.0222 (10)	−0.0016 (8)	0.0053 (8)	0.0011 (7)
N5	0.0408 (13)	0.0244 (11)	0.0416 (13)	−0.0077 (10)	0.0102 (10)	−0.0075 (9)
Ni1	0.0263 (2)	0.0140 (2)	0.0186 (2)	−0.00180 (17)	0.00564 (16)	−0.00025 (16)
O1	0.0322 (10)	0.0267 (9)	0.0246 (9)	−0.0036 (8)	0.0101 (8)	−0.0021 (7)
O2	0.0288 (10)	0.0329 (10)	0.0228 (9)	0.0044 (8)	0.0054 (7)	−0.0001 (7)
O3	0.0420 (11)	0.0222 (9)	0.0288 (10)	−0.0034 (8)	0.0088 (8)	−0.0015 (7)

### Geometric parameters (Å, °)

**Table:**

C1—N1	1.339 (3)	C10—N5	1.331 (3)
C1—C2	1.377 (3)	C10—H10A	0.9300
C1—H1C	0.9300	C11—N5	1.333 (3)
C2—C3	1.390 (3)	C11—C12	1.380 (3)
C2—H2C	0.9300	C11—H11A	0.9300
C3—C4	1.389 (3)	C12—H12A	0.9300
C3—C6	1.465 (3)	N1—Ni1	2.0921 (18)
C4—C5	1.378 (3)	N2—N3	1.364 (3)
C4—H4A	0.9300	Ni1—O2	2.0753 (19)
C5—N1	1.341 (3)	Ni1—O2^i^	2.0753 (19)
C5—H5A	0.9300	Ni1—N1^i^	2.0921 (18)
C6—N2	1.337 (3)	Ni1—O1	2.0945 (17)
C6—N4	1.353 (3)	Ni1—O1^i^	2.0945 (17)
C7—N3	1.334 (3)	O1—H1A	0.85 (3)
C7—N4	1.356 (3)	O1—H1B	0.82 (3)
C7—C8	1.471 (3)	O2—H2A	0.82 (3)
C8—C12	1.384 (3)	O2—H2B	0.82 (3)
C8—C9	1.393 (3)	O3—H3A	0.85 (3)
C9—C10	1.372 (3)	O3—H3B	0.86 (4)
C9—H9A	0.9300		
			
N1—C1—C2	123.3 (2)	C11—C12—C8	119.6 (2)
N1—C1—H1C	118.3	C11—C12—H12A	120.2
C2—C1—H1C	118.3	C8—C12—H12A	120.2
C1—C2—C3	120.1 (2)	C1—N1—C5	116.65 (19)
C1—C2—H2C	119.9	C1—N1—Ni1	122.37 (14)
C3—C2—H2C	119.9	C5—N1—Ni1	120.94 (15)
C4—C3—C2	116.5 (2)	C6—N2—N3	105.95 (17)
C4—C3—C6	122.7 (2)	C7—N3—N2	105.88 (17)
C2—C3—C6	120.7 (2)	C6—N4—C7	101.41 (18)
C5—C4—C3	119.8 (2)	C10—N5—C11	115.9 (2)
C5—C4—H4A	120.1	O2—Ni1—O2^i^	180.0
C3—C4—H4A	120.1	O2—Ni1—N1^i^	90.99 (7)
N1—C5—C4	123.5 (2)	O2^i^—Ni1—N1^i^	89.01 (7)
N1—C5—H5A	118.2	O2—Ni1—N1	89.01 (7)
C4—C5—H5A	118.2	O2^i^—Ni1—N1	90.99 (7)
N2—C6—N4	113.30 (19)	N1^i^—Ni1—N1	180.0
N2—C6—C3	121.29 (19)	O2—Ni1—O1	90.36 (8)
N4—C6—C3	125.27 (19)	O2^i^—Ni1—O1	89.64 (8)
N3—C7—N4	113.45 (19)	N1^i^—Ni1—O1	88.45 (7)
N3—C7—C8	121.77 (19)	N1—Ni1—O1	91.55 (7)
N4—C7—C8	124.7 (2)	O2—Ni1—O1^i^	89.64 (8)
C12—C8—C9	116.5 (2)	O2^i^—Ni1—O1^i^	90.36 (8)
C12—C8—C7	122.2 (2)	N1^i^—Ni1—O1^i^	91.55 (7)
C9—C8—C7	121.3 (2)	N1—Ni1—O1^i^	88.45 (7)
C10—C9—C8	119.6 (2)	O1—Ni1—O1^i^	180.0
C10—C9—H9A	120.2	Ni1—O1—H1A	117 (2)
C8—C9—H9A	120.2	Ni1—O1—H1B	115 (2)
N5—C10—C9	124.3 (2)	H1A—O1—H1B	104 (3)
N5—C10—H10A	117.8	Ni1—O2—H2A	128 (2)
C9—C10—H10A	117.8	Ni1—O2—H2B	119 (2)
N5—C11—C12	124.1 (2)	H2A—O2—H2B	103 (3)
N5—C11—H11A	118.0	H3A—O3—H3B	106 (3)
C12—C11—H11A	118.0		
			
N1—C1—C2—C3	−0.5 (4)	C4—C5—N1—Ni1	178.32 (19)
C1—C2—C3—C4	1.7 (3)	N4—C6—N2—N3	−0.5 (3)
C1—C2—C3—C6	−175.6 (2)	C3—C6—N2—N3	175.4 (2)
C2—C3—C4—C5	−1.7 (4)	N4—C7—N3—N2	−0.2 (3)
C6—C3—C4—C5	175.6 (2)	C8—C7—N3—N2	177.4 (2)
C3—C4—C5—N1	0.5 (4)	C6—N2—N3—C7	0.4 (2)
C4—C3—C6—N2	179.4 (2)	N2—C6—N4—C7	0.4 (3)
C2—C3—C6—N2	−3.4 (3)	C3—C6—N4—C7	−175.3 (2)
C4—C3—C6—N4	−5.2 (4)	N3—C7—N4—C6	−0.2 (3)
C2—C3—C6—N4	172.1 (2)	C8—C7—N4—C6	−177.6 (2)
N3—C7—C8—C12	−172.1 (2)	C9—C10—N5—C11	1.0 (4)
N4—C7—C8—C12	5.1 (4)	C12—C11—N5—C10	−0.2 (4)
N3—C7—C8—C9	4.9 (4)	C1—N1—Ni1—O2	−142.48 (19)
N4—C7—C8—C9	−177.8 (2)	C5—N1—Ni1—O2	40.10 (19)
C12—C8—C9—C10	0.6 (4)	C1—N1—Ni1—O2^i^	37.52 (19)
C7—C8—C9—C10	−176.6 (2)	C5—N1—Ni1—O2^i^	−139.90 (19)
C8—C9—C10—N5	−1.2 (5)	C1—N1—Ni1—N1^i^	139 (100)
N5—C11—C12—C8	−0.4 (4)	C5—N1—Ni1—N1^i^	−39 (100)
C9—C8—C12—C11	0.2 (4)	C1—N1—Ni1—O1	−52.15 (19)
C7—C8—C12—C11	177.3 (2)	C5—N1—Ni1—O1	130.43 (19)
C2—C1—N1—C5	−0.7 (4)	C1—N1—Ni1—O1^i^	127.85 (19)
C2—C1—N1—Ni1	−178.26 (18)	C5—N1—Ni1—O1^i^	−49.57 (19)
C4—C5—N1—C1	0.8 (4)		

Symmetry codes: (i) −*x*+1, −*y*+1, −*z*+1.

### Hydrogen-bond geometry (Å, °)

**Table:**

*D*—H···*A*	*D*—H	H···*A*	*D*···*A*	*D*—H···*A*
O2—H2A···O3^ii^	0.82 (3)	2.03 (3)	2.818 (3)	160 (3)
O3—H3A···N4^iii^	0.85 (3)	2.09 (3)	2.939 (3)	172 (3)
O3—H3B···N5^iv^	0.86 (4)	1.94 (4)	2.789 (3)	173 (3)

Symmetry codes: (ii) −*x*, −*y*+1, −*z*+1; (iii) *x*, −*y*+1/2, *z*+1/2; (iv) −*x*, −*y*, −*z*+1.
